# Do Patients with Luminal A Breast Cancer Profit from Adjuvant Systemic Therapy? A Retrospective Multicenter Study

**DOI:** 10.1371/journal.pone.0168730

**Published:** 2016-12-19

**Authors:** Joachim Diessner, Manfred Wischnewsky, Maria Blettner, Sebastian Häusler, Wolfgang Janni, Rolf Kreienberg, Roland Stein, Tanja Stüber, Lukas Schwentner, Catharina Bartmann, Achim Wöckel

**Affiliations:** 1 Department for Obstetrics and Gynecology, University of Würzburg Medical School, Würzburg, Germany; 2 Faculty of Mathematics and Computer Science, University of Bremen Bremen, Germany; 3 Institut für Medizinische Biometrie, Epidemiologie und Informatik (IMBEI), University of Mainz, Mainz, Germany; 4 Department for Obstetrics and Gynecology, University of Ulm Medical School Ulm, Germany; University of North Carolina at Chapel Hill School of Medicine, UNITED STATES

## Abstract

**Background:**

Luminal A breast cancers respond well to anti-hormonal therapy (HT), are associated with a generally favorable prognosis and constitute the majority of breast cancer subtypes. HT is the mainstay of treatment of these patients, accompanied by an acceptable profile of side effects, whereas the added benefit of chemotherapy (CHT), including anthracycline and taxane-based programs, is less clear-cut and has undergone a process of critical revision.

**Methods:**

In the framework of the BRENDA collective, we analyzed the benefits of CHT compared to HT in 4570 luminal A patients (pts) with primary diagnosis between 2001 and 2008. The results were adjusted by nodal status, age, tumor size and grading.

**Results:**

There has been a progressive reduction in the use of CHT in luminal A patients during the last decade. Neither univariate nor multivariate analyses showed any statistically significant differences in relapse free survival (RFS) with the addition of CHT to adjuvant HT, independent of the nodal status, age, tumor size or grading. Even for patients with more than 3 affected lymph nodes, there was no significant difference (univariate: p = 0.865; HR 0.94; 95% CI: 0.46–1.93; multivariate: p = 0.812; HR 0.92; 95% CI: 0.45–1.88).

**Conclusions:**

The addition of CHT to HT provides minimal or no clinical benefit at all to patients with luminal A breast cancer, independent of the RFS-risk. Consequently, risk estimation cannot be the initial step in the decisional process. These findings–that are in line with several publications–should encourage the critical evaluation of applying adjuvant CHT to patients with luminal A breast cancer.

## Introduction

In 2000, the National Institutes of Health Consensus Development Conference announced that women with early breast cancer profit from cytotoxic chemotherapy as it reduces the risk of recurrence and increases median overall survival [1, 2]. Because of missing data concerning the response of different intrinsic breast cancer subtypes to adjuvant therapy, polychemotherapy was applied to most affected women with localized breast cancer irrespective of menopausal status, affected lymph nodes, tumor subtype or hormone receptor status [1–3]. These guidelines did not consider the heterogeneity of tumor biology or large variety of clinical parameters when developing the best therapy strategy.

During the last decade, recent studies demonstrated that breast cancer is a heterogeneous disease with variable subtypes and different gene array profiles [4–7]. The use of DNA microarrays helped to define molecular subtypes of breast cancer that differ in terms of epidemiology, natural history, dissemination patterns to distant sites, prognosis and response to various therapies [8–14]. The St Gallen panel of 2011 accepted the definition of five different, molecular breast cancer subtypes for clinical application with the intention of identifying which subgroups of breast cancer patients benefit from adjuvant polychemotherapy and those that won’t [15].

Luminal A is a common molecular subtype of breast cancer with a distinct gene expression [16, 17]. This subgroup has the best prognosis and is characterized by high expression of hormone receptors [9], and low expression of the cell-growth marker Ki67 and the human growth factor HER2 [15, 18]. It is well accepted that patients with luminal A tumors strongly benefit from an adjuvant endocrine therapy [15]. The indication of cytotoxic chemotherapy for luminal A patients lies however at the heart of an ongoing debate. On one hand, it is well established that luminal A patients benefit less from cytotoxic therapy than patients with hormone receptor negative or HER2 positive tumors. On the other hand, there seems to be a subset of hormone receptor positive luminal A tumor patients for whom the application of an adjuvant chemotherapy seems to be beneficial [16, 19]. The increasing use of gene expression assays for patients with luminal tumors underlines the high value of clearly identifying subgroups of breast cancer patients who can expect an excellent clinical outcome without the addition of cytotoxic therapy [16, 20, 21]. Right now, the decision for the addition of chemotherapy to adjuvant endocrine therapy for luminal A patients is—next to gene expression assays—mostly influenced by clinical parameters such as tumor size, nodal status, grading, age and indices of cell proliferation. However, factors influencing chemotherapy sensitivity are still poorly understood. For this reason, the intention of this study was to discuss the benefit of cytotoxic therapy for women with luminal A breast cancer depending on different clinical parameters like the number of affected lymph nodes based on the BRENDA data and to demonstrate the therapeutic strategies of the last decade.

## Methods

### Brenda

The BRENDA (= breast cancer care under evidence-based guidelines) collective included patients with breast cancer from the Department of Gynaecology and Obstetrics at the University of Ulm and from 16 partner clinics in Germany for the period 2001–2008. The exact conditions and inclusion criteria of BRENDA have been described previously [22, 23]. For this retrospective study, we extracted data from 4570 patients with luminal A tumors.

### Surrogate Definition

To define the biological breast cancer subtypes, cell proliferation marker Ki67 is currently used. As this marker was not determined for the BRENDA database we modified St. Gallen molecular subtypes as suggested by Parise et al., von Minckwitz et al. and Lips et al. depending on the characteristics of the hormone receptor expression (HR), HER2 overexpression and tumour grade (low = tumour grade of 1 or 2; high = tumour grade of 3) instead: Luminal A is defined by HR positive, HER2 negative- and low tumor grade [24–26].

### Menopausal status

For the definition of the menopausal status we we classified three groups of women who can safely be considered postmenopausal: 1. all women older than 60 years of age; 2. women who underwent a bilateral ovariectomy and 3. women younger than 60 years not using oral contraceptives or hormone replacement therapy (HRT) with an intact uterus and being amenorrhoic for at least one year prior to the diagnosis of breast cancer.

Women having regular menses/periods without using oral contraceptives or HRT are classified as premenopausal.

### Statistical analysis

All categorical data were described using numbers and percentages. Comparisons of categorical variables between groups were made using χ2 tests. Quantitative data were presented using median and range or mean and standard deviations. The primary endpoint was relapse-free survival (RFS), which was assessed by a standard survival analysis using the non-parametric Kaplan-Meier approach. Relapse–free survival is defined as any disease recurrence (local, regional, or distant), but death is censored (not included). If a patient was lost to follow-up, data were censored at the date of the last known contact. When no information was available, the status was coded as missing data. Survival distributions and median survival times were estimated using the Kaplan–Meier product-limit method. The log rank-test was used to provide a formal statistical assessment of the differences between treatment arms. The 5 and 10-year survival rates with 95% confidence interval (95% CI) were computed using Kaplan-Meier product-limit survival probabilities at the specified time points. The Cox proportional hazards model adjusted for age, tumor size etc. was used to estimate the hazard ratio (HR) and 95% confidence intervals. A test of the PH assumption was performed for each covariate and globally using a formal significance test based on the unscaled and scaled Schoenfeld residuals. All statistical tests were two-sided. The level of statistical significance was set at a P-value of 0.05. Statistical analyses were carried out with SPSS [22] and NCSS [10].

### Compliance with Ethical Standards

The Ethics Committee of the University of Ulm, which covers all participating breast cancer centers of the BRENDA network, approved this study and the BRENDA project. Informed consent was documented by the use of a written consent form approved by the BRENDA Review Board and signed by the patient or the patient's legally authorized representative. A copy was given to the person signing the form.

## Results

### Basic characteristics

This study includes 4570 breast cancer patients with the luminal A subtype. 3003 (65.7%) of them had T1 tumors, 1407 (30.8%) had T2, and 1151 (3.3%) T3, and only 9 (0.2%) had T4 tumors. 3122 (68.3%) had no affected lymph nodes, while 963 (21.1%) patients were diagnosed with one to three affected lymph nodes; 485 (10.6%) patients had more than three affected lymph nodes. The median age was 62 (range 25–80). 325 (7.1%) patients received neither chemotherapy nor anti-hormonal therapy (adjuvant systemic therapy = AST), 2712 (59.3) were treated with an adjuvant HT. CHT alone was administered to 157 (3.4%) patients. The combination of hormone and chemotherapy was applied to 1376 (30.1%) patients. The 5-year RFS was 94.3% (mean RFS 10.4y) and the 5-year overall survival (OAS) was 93.2% (mean OAS 10.2y) (Table 1).

**Table 1:** Basic characteristics of the study cohort.

Luminal A breast cancer patients		nodal status			p-value
		Total	negative	positive	
		4570	3122 (68.3%)	1448 (31.7%)	
**Age at primary diagnosis**		mean: 60.9 (SD 11.0) (median:62)	mean: 61.2 (SD 10.9) (median:63)	mean: 60.4 (SD 11.4)	0.027
				(median: 62)	
		Range: 25–80	Range: 25–80	Range:27–80	
**Menopausal status**	premenopausal	941 (20.6)	605 (64.3)	336 (35.7)	0.011
	perimenopausal	138 (3.0)	93 (67.4)	45 (32.6)	
	postmenopausal	3491 (76.4)	2424 (69.4)	1067 (30.6)	
**Grading**	1	672 (14.7)	550 (81.8)	122 (18.2)	< 0.001
	2	3898 (85.3)	2572 (66.0)	1326 (34.0)	
**Tumor size**	T1	3003 (65.7)	2337 (77.8)	666 (22.2)	< 0.001
	T2	1407 (30.8)	739 (52.5)	668 (47.5)	
	T3/T4	160 (3.5)	46 (28.7)	114 (71.3)	
**Guideline adherence**	100% adherent	2876 (62.9)	2140 (74.4)	736 (25.6)	< 0.001
	non-adherent	1694 (37.1)	982 (58.0)	712 (42.0)	

In terms of anti-hormonal therapy 1521(33.3%) patients received aromatase inhibitors, 1020 (67.1%) alone, 501 (32.9%) in combination with chemotherapy. 191 (4.2%) patients were treated with GnRH analogues and 2374 (51.9%) obtained Tamoxifen (TAM), 1573 (66.3%) alone and 801 (33.7%) in combination with chemotherapy. Focused on the group of postmenopausal patients: 1269 (73.9%) patients were treated with Tamoxifen alone and 448 (26.1%) received a combination of Tamoxifen and chemotherapy.

### Clinical parameters influencing the decision making progress for Adjuvant Systemic Therapy (AST)

#### AST and number of affected lymph nodes

**Fig 1:**
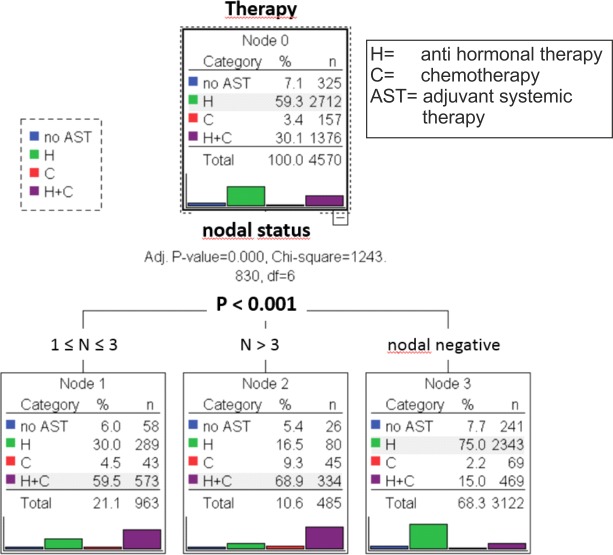
Tree-based classification model of luminal A patients (n = 4570) demonstrates the application of Adjuvant Systemic Therapy (AST), Hormonal Therapy (H) and Chemotherapy (C) depending on the number of affected lymph nodes.

First, we created a classification tree that predicts the application of AST based on the number of affected lymph nodes. This model showed a highly significant difference (p<0.001) for the use of AST and the number of affected lymph nodes. With a negative nodal status, most patients (75.0%) got HT, while 15% received HT and CHT and only 2.2% CHT. 7.7% did not get any AST. In contrast, 68.9% of the patients with more than three affected lymph nodes were treated with a combination of CHT and HT. Here, 16.5% of the patients got only HT, 9.3% CHT and 5.4% no AST. The combination of CHT and HT was applied to 59.5% of patients with one to three affected lymph nodes, while 30.0% of them got a HT, 4.5% only a CHT and 6.0% no AST (Fig 1).

#### AST and age

A model that predicts the application of AST based on the age at first diagnosis demonstrated a highly significant adverse correlation (p<0.001) between different age groups and the administered AST. Most patients younger than 35 years (65.2%), got both, CHT and HT, whereas 15.2% received only CHT, 10.9% HT and 8.7% no AST. For patients between 35 and 65 years of age, we found 49.7% with HT, 39.7% with CHT and HT, 6.2% with no AST and 4.4% with CHT. Most of the young elderly patients between 65 and 75 years (69.7%) got HT, 21.5% CHT and HT, 6.7% no AST and 2.2% only CHT. In the group of the elderly over 75 years, 82.6% received HT, 12.6% no AST, 3.6% CHT and HT and only 1.1% CHT.

#### AST and grading

Another highly significant (p<0.001) classification tree constitutes the use of AST and grading. HT was applied to 77.2% of the patients with G1 tumors and 56.2% of patients with G2 tumors. A combination of CHT and HT was used in 13.5% of patients with G1 tumors, but in 32.9% of patients with G2 tumors. CHT alone was administered to 1.3% of patients with G1 tumors and to 3.8% of patients with G2 tumors. No AST was used after the diagnosis of a G1 or G2 tumor for 7.9% and 7.0% of the patients, respectively.

#### AST and tumor size

**Fig 2:**
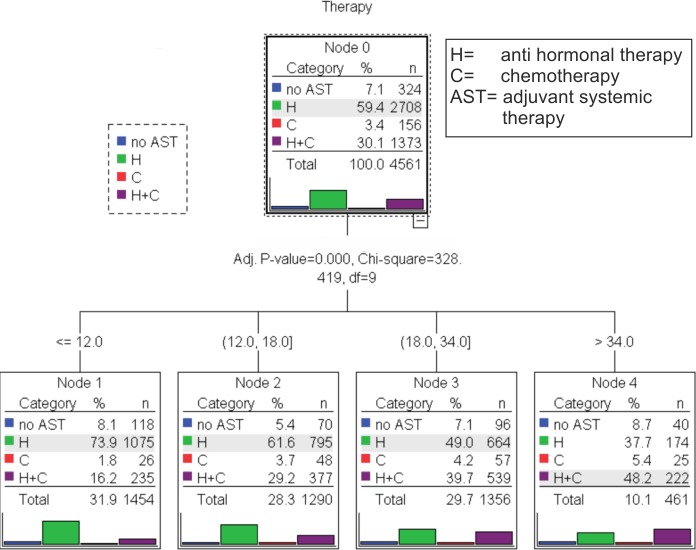
Tree-based classification model luminal A patients (n = 4570) demonstrates the application of Adjuvant Systemic Therapy (AST), Hormonal Therapy (H) and Chemotherapy (C) depending on the size of the primary tumor in millimetres.

Another decision tree model showed that tumor size had a strong impact on the application of AST (p<0.001). For patients with T1 tumors, 67.0% received HT, 23.2% CHT and HT, 2.8% CHT and 7.0% no AST, whereas 46.2% of the patients with T2 tumors got HT, 46.2% CHT and HT, 7.4% no AST and 4.4% CHT. 55.6% of the patients with T3 tumors had CHT and HT, 31.1% HT, 6.6% CHT and 6.6% no AST. Fig 2 shows optimal cut points for the tumor size and a highly significant difference (p<0.001). Here, we have used the following classification: ≤12mm; 12-18mm; 18-34mm and >34mm (Fig 2).

#### AST, nodal status, age, grading and tumor size

**Fig 3:**
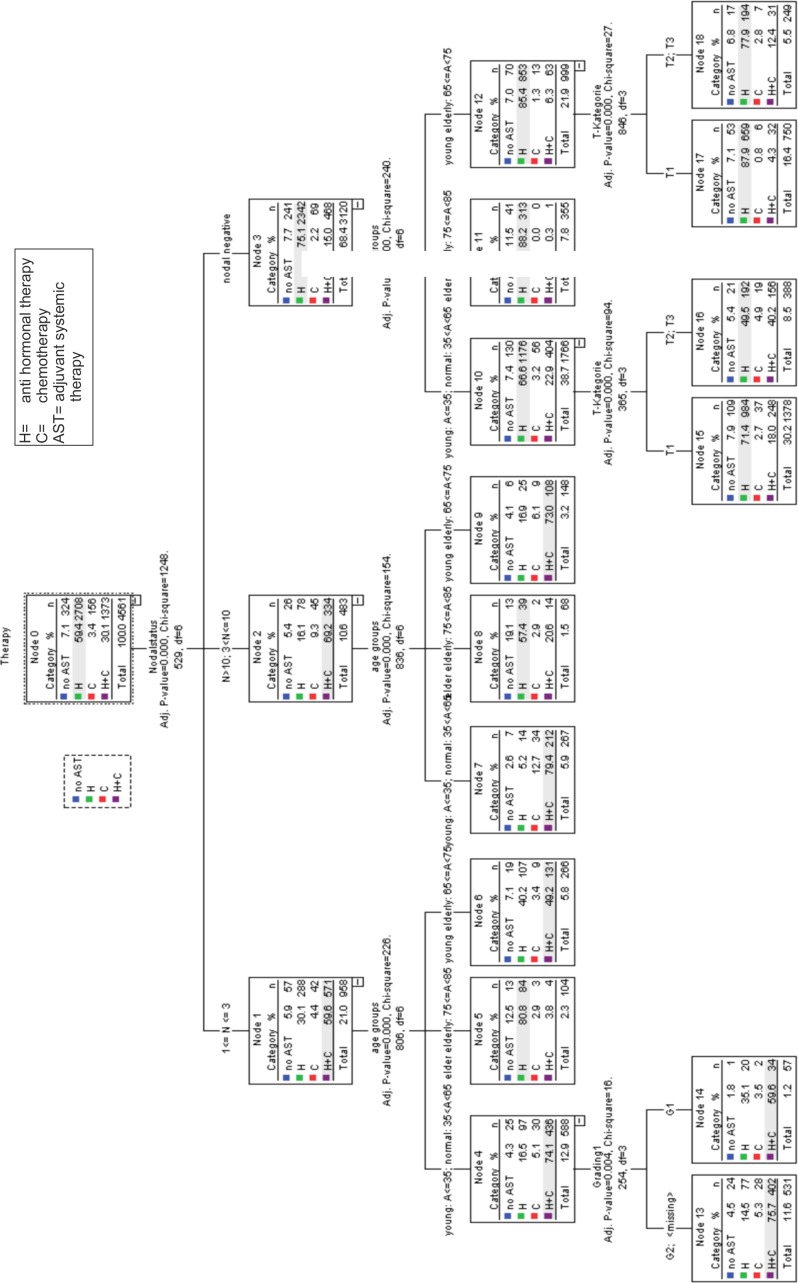
Tree-based classification model luminal A patients (n = 4570) demonstrates the therapeutical algorithm concerning Adjuvant Systemic Therapy (AST), Hormonal Therapy (H) and Chemotherapy (C) depending on nodal status, grading (G1,G2), age and tumor size.

Fig 3 summarizes a branched tree-based classification model of the significant differences in AST depending on nodal status, age, grading and tumor size (Fig 3).

#### AST and year of diagnosis

Cross tabulation analysis showed a highly significant reduction (p<0.001) in chemotherapy for patients with luminal A nodal negative breast cancer from 2001 to 2008. In 2001, 28.8% of them received CHT and HT, 5.5% only CHT. In contrast, in 2008, only 12.1% got CHT and HT and 2.0% CHT (Table 2). There was no significant reduction (p = 0.332) in the use of chemotherapy for node-positive luminal A breast cancer patients in this period. CHT and HT were applied to 67.6% of the patients in 2001, to 60.8% in 2008. CHT alone was applied to 2.9% of the patients in 2001 and to 6.6% in 2008 (Table 3).

**Table 2:** Cross tabulation of the year of diagnosis and the therapy for luminal A nodal negative patients (Pearson chi square: p<0.001).

Luminal A nodal negative patients		Therapy			
		no AST	ET	CHT	ET+CHT
**year of primary diagnosis**	**2001**	8.2%	57.5%	5.5%	28.8%
	**2002**	13.9%	65.3%	3.0%	17.8%
	**2003**	7.0%	70.6%	3.1%	19.3%
	**2004**	8.1%	66.1%	3.0%	22.7%
	**2005**	5.6%	78.2%	2.0%	14.2%
	**2006**	8.7%	77.1%	2.3%	12.0%
	**2007**	8.8%	79.9%	0.8%	10.5%
	**2008**	6.9%	79.0%	2.0%	12.1%
**Total**		**7.7%**	**75.0%**	**2.2%**	**15.0%**

**Table 3:** Cross tabulation of the year of diagnosis and the therapy for luminal A nodal positive patients (Pearson chi square: p = 0.332).

Luminal A nodal positive patients		Therapy			
		no AST	ET	CHT	ET+CHT
**year of primary diagnosis**	**2001**	5.9%	23.5%	2.9%	67.6%
	**2002**	2.6%	15.8%	7.9%	73.7%
	**2003**	5.9%	23.4%	6.4%	64.4%
	**2004**	8.2%	29.1%	5.1%	57.7%
	**2005**	4.7%	33.2%	4.3%	57.8%
	**2006**	5.7%	23.6%	6.1%	64.6%
	**2007**	5.4%	19.1%	7.9%	67.6%
	**2008**	5.9%	26.7%	6.6%	60.8%
**Total**		**5.8%**	**25.5%**	**6.1%**	**62.6%**

### Clinical benefit of AST for luminal A patients

Firstly, we examined RFS of all luminal A patients stratified by nodal status. As expected, the best RFS could be observed for patients with no affected lymph nodes, followed by patients with 1–3 affected lymph nodes (HR 1.73; 95% CI: 1.26–2.39; p = 0.001) and patients with the 4–10 affected lymph nodes (HR 3.25; 95% CI: 2.21–4.79; p<0.001). Patients with more than 10 affected lymph nodes had the worst RFS (HR 8.35; 95% CI: 5.67–12.29; p<0.001) compared to N0 luminal A patients. Similar results were obtained for OAS. The next best OAS was observed for patients with 1–3 affected lymph nodes (HR 1.55; 95% CI: 1.18–2.05; p = 0.002) compared to patients with no affected lymph nodes, followed by patients with 4–10 affected lymph nodes (HR 2.76; 95% CI: 1.96–3.88; p<0.001). The worst OAS was observed for patients with more than 10 affected lymph nodes (HR 5.83; 95% CI: 4.04–8.43; p<0.001). For N0 (N1)-patients the 5-year RFS was 96.2% (90.2%) and the 5-year OAS was 94.9% (89.2%).

**Fig 4:**
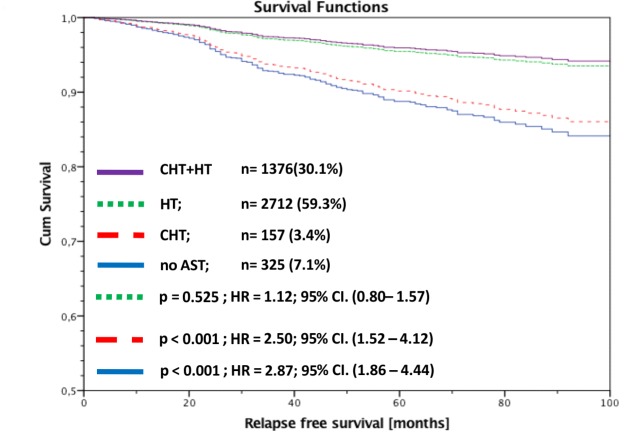
RFS of luminal A patients stratified by adjuvant systemic therapy and adjusted by nodal status.

Secondly, we analysed RFS of all luminal A patients stratified by AST and adjusted by nodal status. Patients with HT alone had no significant worse outcome compared to patients with CHT and HT (HR 1.12; 95% CI: 0.80–1.57; p = 0.525). In contrast to this result, patients with CHT alone or no AST had a highly significant worse outcome compared to patients with CHT+HT (CHT alone: HR 2.50; 95% CI: 1.52-.4.12; p<0.001; no AST: HR 2.87; 95% CI: 1.86–4.44; p<0.001). There was no significant difference between patients with CHT alone and no AST (p = 0.988) (Fig 4). Because of the more powerful HT including aromatase inhibitors (AI) we made a differentiated analysis for TAM and AI each of these in combination with CHT. These RFS data were adjusted by menopausal status. AI vs AI + CHT (RFS: p = 0.373; HR = 0.78; 95% CI (0.46–1.34); TAM vs TAM+CHT (RFS: p<0.001; HR = 0.46; 95% CI (0.32–0.68); postmenopausal: TAM vs TAM+CHT (RFS: p = 0.002; HR = 0.49; 95% CI(0.31–0.77) The analysis could once again not detect any clinical benefit for the addition of CHT to HT, neither for TAM nor for AI. Quite contrary TAM alone shows even better RFS data than the combination of TAM and CHT even for postmenopausal women.

Finally, we analyzed RFS of luminal A breast cancer patients depending on different AST and different nodal status adjusted by age, tumor size and grading (multivariate). The univariate and multivariate results are presented in Table 4.

**Table 4:** Summarized presentation of the clinical outcome of breast cancer patients depending on nodal status and AST adjusted by age, grading and tumor size.

reference: CHT+HT		univariate			multivariate*		
		HR	95% CI	p-value	HR	95% CI	p-value
**N0 (n = 3122; 68.3%)**	HT	0.98	0.57–1.69	0.937	1.1	0.63–1.91	0.742
	CHT alone	1.83	0.61–5.47	0.28	1.89	0.63–5.67	0.254
	no AST	2.73	1.39–5.36	0.004	2.89	1.45–5.77	0.003
**1–3 affected lymph nodes (n = 963; 21.1%)**	HT	1.57	0.87–2.85	0.134	1.57	0.87–2.84	0.137
	CHT alone	3.96	1.63–9.65	0.002	3.95	1.62–9.62	0.002
	no AST	2.99	1.23–7.29	0.016	2.98	1.23–7.26	0.016
**3 affected lymph nodes (n = 485; 10.6%)**	HT	0.94	0.46–1.93	0.865	0.92	0.45–1.88	0.812
	CHT alone	2.08	1.02–4.27	0.046	2.42	1.16–5.01	0.018
	no AST	2.03	0.98–5.41	0.055	2.24	0.96–5.27	0.064

* adjusted by age, tumor size and grading

**Fig 5:**
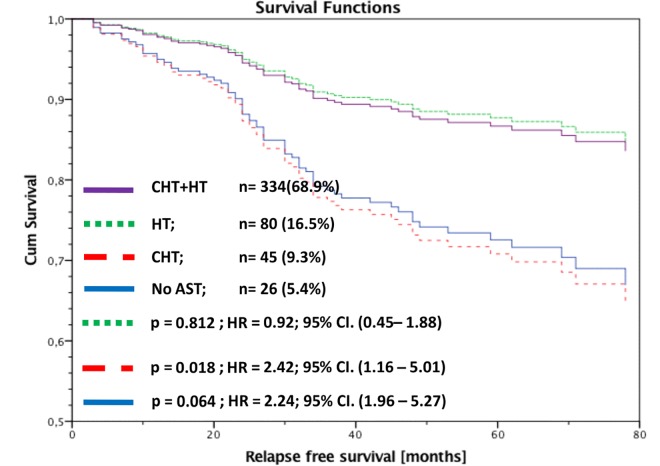
RFS of luminal A patients with more than 3 affected lymph nodes stratified by adjuvant systemic therapy and adjusted by age, tumor size and grading.

In none of the corresponding subgroups, with respect to nodal status, could we find a significant difference in RFS between patients with CHT+HT and HT alone. Also, for patients with more than 3 affected lymph nodes there was no significant difference in RFS between HT alone and CHT+HT (univariate: HR = 0.94; 95% CI: 0.46–1.93; p = 0.865; multivariate: HR = 0.92; 95% CI: 0.45–1.88; p = 0.812)) (Fig 5).

## Discussion

Today, it is well accepted that genomic tumor subtypes as well as different clinical parameters influence the adjuvant therapeutic strategy of breast cancer patients [4]. There are different breast cancer subtypes and clinical parameters that make the application of cytotoxic therapy indispensable [15]. For the subgroup of luminal A breast cancer, the question of the clinical benefit of the application of CHT cannot be answered unequivocally.

As the administration of chemotherapy involves considerable physical and emotional stress for patients, the application should at least improve RFS and OAS of the affected women significantly. Therefore, the question of whether patients are optimally treated without cytostatics should be answered clearly.

The recommendations of the National Institutes of Health Consensus Development Conference in 2000 for CHT for all early breast cancer patients have been overtaken by the identification of different biological behavior of the intrinsic breast cancer subtypes [1, 2]. We could demonstrate a highly significant reduction (p<0.001) in the use of chemotherapy in patients with luminal A nodal negative breast cancer from 2001 to 2008.

This trend matches the performed research and is in line with the literature [12, 16, 26].

However, the practice of omitting chemotherapy for women with luminal A tumors–independently of clinical parameters–seems to be controversial. The strategy to waive chemotherapy was based on several meta-analyses that found no added benefit of CHT for luminal A tumors [1, 27]. Most of these studies applied CMF-based chemotherapy and prescribed Tamoxifen concurrently with CHT. This therapeutical setting is however no longer in line with breast cancer management guidelines. We therefore performed subgroup analysis in the BRENDA database for luminal A patients in the period from 2001–2008.

We discovered that nodal status, grading, age and tumor size were highly significant indicators for the application of CHT in patients with luminal A tumors. Especially, nodal status seems to play an important role for the application of CHT.

We could illustrate that patients with node-negative luminal A tumors—independent of the menopausal status and of the type of anti-hormonal therapy- did not benefit in terms of RFS from an addition of CHT to HT. These findings are in line with the results published by the IBCSG (International Breast Cancer Study Group) IX trial that demonstrated in 2011 that postmenopausal women with node-negative luminal A disease do not benefit from adding cyclophosphamide, methotrexate, and fluorouracil to HT [28]. The study IBCSG VIII that focused on premenopausal, nodal negative luminal A tumors also showed strong evidence that chemotherapy has no added clinical benefit when compared to endocrine therapy alone [29, 30].

For nodal positive luminal A tumors, we could again not find any clinical benefit in terms of RFS by adding CHT to HT. These results are in contrast to the findings published by Albain et al. for postmenopausal, node-positive luminal A tumors in the clinical trial SWOG 8814. This analysis demonstrated that the adjuvant treatment with a combination of CAF plus Tamoxifen significantly improved disease-free survival, as compared to Tamoxifen alone [31]. It has to be mentioned, that the anti-hormonal therapy in this analysis was limited to Tamoxifen and did not integrate the positive effect of aromatase inhibitors for postmenopausal women [32].

On the other hand, there are several retrospective analyses that are in line with our results.

Nielsen et al. could not find a 10-year disease-free survival difference between premenopausal women with luminal A, lymph node-positive invasive breast cancer, larger than 5 cm who received chemotherapy and those who did not [18, 33]. This analysis focuses however on a study cohort between 1977 and 1983, whereby none of the patients got adjuvant HT. Therefore, this trial does not mirror the current standard of care.

Another study on the effect of chemotherapy for luminal A tumors was published in 2013 by Uchida et al. This analysis could not detect a significant difference in RFS between patients with tumor size greater than 2 cm and/or positive lymph node status who either received chemotherapy or not [34]. It has to be added that this study group used a less-efficacious chemotherapy regimen with regard to dosages. Moreover, the retrospective analysis published by Uchida et al. contained only 24 patient, whereas we extracted data from 4570 luminal A tumor patients.

A further publication that focused on the questionable use of CHT for luminal A, nodal positive patients was published in 2015 by Kwak et al. Kwak and co-workers demonstrated in a multivariable analysis no significant benefit of adjuvant CHT for disease free survival for patients with luminal A T1-2 nodal positive tumors [35]. We could confirm the results of this analysis even for the subgroups of nodal positive (1–3 affected lymph nodes) and highly nodal positive (more than three affected lymph nodes) patients.

In our BRENDA collective 21% of the luminal A patients had 1–3 and 10.6% more than 3 affected lymph nodes. Comparing our data with the literature shows a large variety for the number of affected lymph nodes. Uchida et al. published in 2013 a study cohort with 26% of luminal A patients with lymph node involvement [34]. Han et al. analyzed a population of 44% of luminal A patients with affected lymph nodes [36], whereas de Oliveira Filho et al. showed data that are very much in line with our results of 32% of luminal A patients and lymph node metastasis [37]. Of course, lymph node involvement is a predictive factor for the clinical outcome of luminal A breast cancer patients. In our data, we analyzed that relapse free survival an overall survival is significantly influenced by the number of affected lymph nodes (data not shown). We could however not demonstrate a clinical benefit for the addition of CHT to HT independently of the number of affected lymph nodes for luminal A tumors (Fig 4 and Fig 5).

One limitation of this analysis is the renunciation of high dense, high intense chemotherapy. Möbus et al. described that patients with more than three affected lymph nodes benefit from this intense therapy [38]. In the period of 2001–2008, the standard chemotherapy was anthracycline based and did not include high intense regimes. Therefore, further studies are necessary to analyse if there is a clinical benefit of high intense chemotherapy in addition to anti-hormonal therapy for patients with more than three affected lymph nodes and luminal A tumors.

Moreover, there are now advantages for a longer period of endocrine therapy (10 years instead of 5 years) [39, 40]. Therefore, the prolonged endocrine therapy should also be compared to the addition of CHT for luminal A breast cancer patients. It can only be guessed that the effect of CHT could be even lower for patients with luminal A tumors who received long term HT.

A final critical point, that should be mentioned is based on the fact that we focused on RFS in our analysis. Indeed, we also performed OAS analysis. Based on a lack of OAS follow up data we did, however, not get statistically clear results. OAS analysis for luminal A breast cancer patients should, therefore, be performed in an even bigger study cohort.

In conclusion, the decision for the use of CHT in addition to HT for luminal A tumors should not be based on the traditional clinical factors such as tumor size, age, grading and the number of affected lymph nodes.

Based on these clinical parameters, we could not identify a subgroup of luminal A breast cancer patients that had a clear benefit from the application of CHT in addition to HT, independent of the type of anti-hormonal therapy. Regarding the negative acute and long-term side effects of CHT, the application of theses cytotoxic substances should, however, be justified by a significant clinical benefit.

Identifying new predictors of chemo sensitivity constitutes, therefore, an important task in developing tailored therapies for women with luminal A tumors.

## References

[1] Effects of chemotherapy and hormonal therapy for early breast cancer on recurrence and 15-year survival: an overview of the randomised trials. Lancet. 2005;365(9472):1687–717. Epub 2005/05/17. 10.1016/S0140-6736(05)66544-0 15894097

[2] The National Institutes of Health Consensus Development Conference: Adjuvant Therapy for Breast Cancer—Bethesda Maryland, USA: November 1–3, 2000—Proceedings. J Natl Cancer Inst Monogr 5–15, 2001 12083020

[3] Coates AS, Colleoni M, Goldhirsch A. Is adjuvant chemotherapy useful for women with luminal a breast cancer? Journal of clinical oncology: official journal of the American Society of Clinical Oncology. 2012;30(12):1260–3. Epub 2012/02/23. 22355052 10.1200/JCO.2011.37.7879

[4] Perou CM, Sorlie T, Eisen MB, van de Rijn M, Jeffrey SS, Rees CA, et al Molecular portraits of human breast tumours. Nature. 2000;406(6797):747–52. Epub 2000/08/30. 10.1038/35021093 10963602

[5] Prat A, Perou CM. WITHDRAWN: Deconstructing the molecular portraits of breast cancer. Molecular oncology. 2010. Epub 2010/05/08. 10.1016/j.molonc.2010.04.003 20447881

[6] Nielsen TO, Parker JS, Leung S, Voduc D, Ebbert M, Vickery T, et al A comparison of PAM50 intrinsic subtyping with immunohistochemistry and clinical prognostic factors in tamoxifen-treated estrogen receptor-positive breast cancer. Clinical cancer research: an official journal of the American Association for Cancer Research. 2010;16(21):5222–32. Epub 2010/09/15. PubMed Central PMCID: PMC2970720. 20837693 10.1158/1078-0432.CCR-10-1282 PMC2970720

[7] Sorlie T, Perou CM, Tibshirani R, Aas T, Geisler S, Johnsen H, et al Gene expression patterns of breast carcinomas distinguish tumor subclasses with clinical implications. Proceedings of the National Academy of Sciences of the United States of America. 2001;98(19):10869–74. Epub 2001/09/13. PubMed Central PMCID: PMC58566. 10.1073/pnas.191367098 11553815 PMC58566

[8] Geyer FC, Marchio C, Reis-Filho JS. The role of molecular analysis in breast cancer. Pathology. 2009;41(1):77–88. Epub 2008/12/18. 10.1080/00313020802563536 19089743

[9] Phipps AI, Buist DS, Malone KE, Barlow WE, Porter PL, Kerlikowske K, et al Reproductive history and risk of three breast cancer subtypes defined by three biomarkers. Cancer causes & control: CCC. 2011;22(3):399–405. Epub 2010/12/25. PubMed Central PMCID: PMC3042513. 21184265 10.1007/s10552-010-9709-0 PMC3042513

[10] Millikan RC, Newman B, Tse CK, Moorman PG, Conway K, Dressler LG, et al Epidemiology of basal-like breast cancer. Breast cancer research and treatment. 2008;109(1):123–39. Epub 2007/06/21. PubMed Central PMCID: PMC2443103. 10.1007/s10549-007-9632-6 17578664 PMC2443103

[11] Nguyen PL, Taghian AG, Katz MS, Niemierko A, Abi Raad RF, Boon WL, et al Breast cancer subtype approximated by estrogen receptor, progesterone receptor, and HER-2 is associated with local and distant recurrence after breast-conserving therapy. Journal of clinical oncology: official journal of the American Society of Clinical Oncology. 2008;26(14):2373–8. Epub 2008/04/17. 18413639 10.1200/JCO.2007.14.4287

[12] Hugh J, Hanson J, Cheang MC, Nielsen TO, Perou CM, Dumontet C, et al Breast cancer subtypes and response to docetaxel in node-positive breast cancer: use of an immunohistochemical definition in the BCIRG 001 trial. Journal of clinical oncology: official journal of the American Society of Clinical Oncology. 2009;27(8):1168–76. Epub 2009/02/11. PubMed Central PMCID: PMC2667821. 19204205 10.1200/JCO.2008.18.1024 PMC2667821

[13] Liedtke C, Mazouni C, Hess KR, Andre F, Tordai A, Mejia JA, et al Response to neoadjuvant therapy and long-term survival in patients with triple-negative breast cancer. Journal of clinical oncology: official journal of the American Society of Clinical Oncology. 2008;26(8):1275–81. Epub 2008/02/06. 18250347 10.1200/JCO.2007.14.4147

[14] Wo JY, Taghian AG, Nguyen PL, Raad RA, Sreedhara M, Bellon JR, et al The association between biological subtype and isolated regional nodal failure after breast-conserving therapy. International journal of radiation oncology, biology, physics. 2010;77(1):188–96. Epub 2010/02/23. 10.1016/j.ijrobp.2009.04.059 20171798

[15] Goldhirsch A, Wood WC, Coates AS, Gelber RD, Thurlimann B, Senn HJ. Strategies for subtypes—dealing with the diversity of breast cancer: highlights of the St. Gallen International Expert Consensus on the Primary Therapy of Early Breast Cancer 2011. Annals of oncology: official journal of the European Society for Medical Oncology / ESMO. 2011;22(8):1736–47. Epub 2011/06/29. PubMed Central PMCID: PMC3144634. 10.1093/annonc/mdr304 PMC3144634 21709140

[16] Lim E, Winer EP. Adjuvant chemotherapy in luminal breast cancers. Breast. 2011;20 Suppl 3:S128–31. Epub 2011/11/02. 22015279 10.1016/S0960-9776(11)70309-5

[17] Sorlie T, Tibshirani R, Parker J, Hastie T, Marron JS, Nobel A, et al Repeated observation of breast tumor subtypes in independent gene expression data sets. Proceedings of the National Academy of Sciences of the United States of America. 2003;100(14):8418–23. Epub 2003/06/28. PubMed Central PMCID: PMC166244. 10.1073/pnas.0932692100 12829800 PMC166244

[18] Ejlertsen B, Mouridsen HT, Jensen MB, Andersen J, Andersson M, Kamby C, et al Cyclophosphamide, methotrexate, and fluorouracil; oral cyclophosphamide; levamisole; or no adjuvant therapy for patients with high-risk, premenopausal breast cancer. Cancer. 2010;116(9):2081–9. Epub 2010/02/27. 10.1002/cncr.24969 20186830

[19] Berry DA, Cirrincione C, Henderson IC, Citron ML, Budman DR, Goldstein LJ, et al Estrogen-receptor status and outcomes of modern chemotherapy for patients with node-positive breast cancer. JAMA: the journal of the American Medical Association. 2006;295(14):1658–67. Epub 2006/04/13. PubMed Central PMCID: PMC1459540. 10.1001/jama.295.14.1658 16609087 PMC1459540

[20] Kuijer A, Drukker CA, Elias SG, Smorenburg CH, Th Rutgers EJ, Siesling S, et al Changes over time in the impact of gene-expression profiles on the administration of adjuvant chemotherapy in estrogen receptor positive early stage breast cancer patients: A nationwide study. International journal of cancer Journal international du cancer. 2016;139(4):769–75. Epub 2016/04/12. 10.1002/ijc.30132 27062369

[21] Thomssen C, Augustin D, Ettl J, Haidinger R, Luck HJ, Luftner D, et al ABC3 Consensus: Assessment by a German Group of Experts. Breast Care (Basel). 2016;11(1):61–70. Epub 2016/04/07. PubMed Central PMCID: PMC4813642. 27051399 10.1159/000443515 PMC4813642

[22] Diessner J, Wischnewsky M, Stuber T, Stein R, Krockenberger M, Hausler S, et al Evaluation of clinical parameters influencing the development of bone metastasis in breast cancer. BMC cancer. 2016;16:307 Epub 2016/05/14. PubMed Central PMCID: PMC4865990. 10.1186/s12885-016-2345-7 27175930 PMC4865990

[23] Diessner J, Van Ewijk R, Weiss CR, Janni W, Wischnewsky MB, Kreienberg R, et al Identifying the impact of inflammatory breast cancer on survival: a retrospective multi-center cohort study. Archives of gynecology and obstetrics. 2015;292(3):655–64. Epub 2015/03/31. 10.1007/s00404-015-3691-4 25814296

[24] Parise CA, Caggiano V. Breast Cancer Survival Defined by the ER/PR/HER2 Subtypes and a Surrogate Classification according to Tumor Grade and Immunohistochemical Biomarkers. Journal of cancer epidemiology. 2014;2014:469251 Epub 2014/06/24. PubMed Central PMCID: PMC4058253. 10.1155/2014/469251 24955090 PMC4058253

[25] von Minckwitz G, Untch M, Blohmer JU, Costa SD, Eidtmann H, Fasching PA, et al Definition and impact of pathologic complete response on prognosis after neoadjuvant chemotherapy in various intrinsic breast cancer subtypes. Journal of clinical oncology: official journal of the American Society of Clinical Oncology. 2012;30(15):1796–804. Epub 2012/04/18. 22508812 10.1200/JCO.2011.38.8595

[26] Lips EH, Mulder L, de Ronde JJ, Mandjes IA, Koolen BB, Wessels LF, et al Breast cancer subtyping by immunohistochemistry and histological grade outperforms breast cancer intrinsic subtypes in predicting neoadjuvant chemotherapy response. Breast cancer research and treatment. 2013;140(1):63–71. Epub 2013/07/06. PubMed Central PMCID: PMC3706735. 10.1007/s10549-013-2620-0 23828499 PMC3706735

[27] Endocrine responsiveness and tailoring adjuvant therapy for postmenopausal lymph node-negative breast cancer: a randomized trial. Journal of the National Cancer Institute. 2002;94(14):1054–65. Epub 2002/07/18. 12122096 10.1093/jnci/94.14.1054

[28] Aebi S, Sun Z, Braun D, Price KN, Castiglione-Gertsch M, Rabaglio M, et al Differential efficacy of three cycles of CMF followed by tamoxifen in patients with ER-positive and ER-negative tumors: long-term follow up on IBCSG Trial IX. Annals of oncology: official journal of the European Society for Medical Oncology / ESMO. 2011;22(9):1981–7. Epub 2011/02/02. PubMed Central PMCID: PMC3202167. 10.1093/annonc/mdq754 PMC3202167 21282282

[29] Viale G, Regan MM, Maiorano E, Mastropasqua MG, Golouh R, Perin T, et al Chemoendocrine compared with endocrine adjuvant therapies for node-negative breast cancer: predictive value of centrally reviewed expression of estrogen and progesterone receptors—International Breast Cancer Study Group. Journal of clinical oncology: official journal of the American Society of Clinical Oncology. 2008;26(9):1404–10. Epub 2008/03/20. 18349391 10.1200/JCO.2007.10.6393

[30] Karlsson P, Sun Z, Braun D, Price KN, Castiglione-Gertsch M, Rabaglio M, et al Long-term results of International Breast Cancer Study Group Trial VIII: adjuvant chemotherapy plus goserelin compared with either therapy alone for premenopausal patients with node-negative breast cancer. Annals of oncology: official journal of the European Society for Medical Oncology / ESMO. 2011;22(10):2216–26. Epub 2011/02/18. PubMed Central PMCID: PMC3179412. 10.1093/annonc/mdq735 PMC3179412 21325445

[31] Albain KS, Barlow WE, Ravdin PM, Farrar WB, Burton GV, Ketchel SJ, et al Adjuvant chemotherapy and timing of tamoxifen in postmenopausal patients with endocrine-responsive, node-positive breast cancer: a phase 3, open-label, randomised controlled trial. Lancet. 2009;374(9707):2055–63. Epub 2009/12/17. PubMed Central PMCID: PMC3140679. 10.1016/S0140-6736(09)61523-3 20004966 PMC3140679

[32] Howell A, Cuzick J, Baum M, Buzdar A, Dowsett M, Forbes JF, et al Results of the ATAC (Arimidex, Tamoxifen, Alone or in Combination) trial after completion of 5 years' adjuvant treatment for breast cancer. Lancet. 2005;365(9453):60–2. Epub 2005/01/11. 10.1016/S0140-6736(04)17666-6 15639680

[33] Nielsen TO JM-B, Gao D, Leung S, Burugu S, Liu S, Tykjær Jørgensen CL, Balslev E, Ejlertsen B. High risk premenopausal luminal A breast cancer patients derive no benefit from adjuvant chemotherapy: Results from DBCG77B randomized trial. Pathology and Laboratory Medicine. 2015;S1–08.

[34] Uchida N, Suda T, Ishiguro K. Effect of chemotherapy for luminal a breast cancer. Yonago acta medica. 2013;56(2):51–6. Epub 2013/09/14. PubMed Central PMCID: PMC3771208. 24031152 PMC3771208

[35] Kwak HY, Chae BJ, Eom YH, Hong YR, Seo JB, Bae JS, et al Is adjuvant chemotherapy omissible in women with T1-2 stage, node-positive, luminal A type breast cancer? J Chemother. 2015;27(5):290–6. Epub 2015/05/15. 10.1179/1973947815Y.0000000015 25974160

[36] Han Y, Li Q, Xu BH, Zhang P, Yuan P, Wang JY, et al Adjuvant chemotherapy may improve survival of patients with luminal A breast cancer and positive lymph nodes. Genetics and molecular research: GMR. 2015;14(3):8563–73. Epub 2015/09/09. 10.4238/2015.July.31.4 26345787

[37] de Oliveira Filho HR, Doria MT, Piato JR, Soares Junior JM, Filassi JR, Baracat EC, et al Criteria for prediction of metastatic axillary lymph nodes in early-stage breast cancer. Revista brasileira de ginecologia e obstetricia: revista da Federacao Brasileira das Sociedades de Ginecologia e Obstetricia. 2015;37(7):308–13. Epub 2015/08/08. 10.1590/S0100-720320150005343 26247250

[38] Mobus V. Adjuvant Dose-Dense Chemotherapy in Breast Cancer: Standard of Care in High-Risk Patients. Breast Care (Basel). 2016;11(1):8–12. Epub 2016/04/07. PubMed Central PMCID: PMC4813643. 27051389 10.1159/000444004 PMC4813643

[39] Mayer EL, Burstein HJ. Adjuvant endocrine therapy for postmenopausal women: Type and duration. Breast. 2015;24 Suppl 2:S126–8. Epub 2015/08/19. 26279133 10.1016/j.breast.2015.07.028

[40] Mayer EL, Burstein HJ. Postmenopausal breast cancer: a best endocrine strategy? Lancet. 2015;386(10001):1317–9. Epub 2015/07/28. 10.1016/S0140-6736(15)61206-5 26211823

